# Nonfermented Dairy Intake, but Not Fermented Dairy Intake, Associated with a Higher Risk of Depression in Middle-Age and Older Finnish Men

**DOI:** 10.1093/jn/nxac128

**Published:** 2022-06-02

**Authors:** Meghan Hockey, Erin Hoare, Mohammadreza Mohebbi, Tommi Tolmunen, Sari Hantunen, Tomi-Pekka Tuomainen, Helen Macpherson, Heidi Staudacher, Felice N Jacka, Jykri K Virtanen, Tetyana Rocks, Anu Ruusunen

**Affiliations:** IMPACT (the Institute for Mental and Physical Health and Clinical Translation), Food & Mood Centre, Deakin University, Geelong, Australia; IMPACT (the Institute for Mental and Physical Health and Clinical Translation), Food & Mood Centre, Deakin University, Geelong, Australia; Faculty of Health, Biostatistics Unit, Deakin University, Geelong, Australia; Department of Adolescent Psychiatry, Kuopio University Hospital, Kuopio, Finland; Institute of Clinical Medicine/Psychiatry, University of Eastern Finland, Kuopio, Finland; Institute of Public Health and Clinical Nutrition, University of Eastern Finland, Kuopio, Finland; Institute of Public Health and Clinical Nutrition, University of Eastern Finland, Kuopio, Finland; Faculty of Health, Institute for Physical Activity and Nutrition, School of Exercise and Nutrition, Deakin University, Geelong, Australia; IMPACT (the Institute for Mental and Physical Health and Clinical Translation), Food & Mood Centre, Deakin University, Geelong, Australia; IMPACT (the Institute for Mental and Physical Health and Clinical Translation), Food & Mood Centre, Deakin University, Geelong, Australia; Centre for Adolescent Health, Murdoch Children's Research Institute, Melbourne, Australia; Black Dog Institute, Sydney, Australia; Institute of Public Health and Clinical Nutrition, University of Eastern Finland, Kuopio, Finland; IMPACT (the Institute for Mental and Physical Health and Clinical Translation), Food & Mood Centre, Deakin University, Geelong, Australia; IMPACT (the Institute for Mental and Physical Health and Clinical Translation), Food & Mood Centre, Deakin University, Geelong, Australia; Institute of Public Health and Clinical Nutrition, University of Eastern Finland, Kuopio, Finland; Department of Psychiatry, Kuopio University Hospital, Kuopio, Finland

**Keywords:** dairy, fermented foods, milk, depression, nutrition, mental health

## Abstract

**Background:**

Despite the putative health benefits of fermented dairy products, evidence on the association between fermented dairy and nonfermented dairy intake, and depression incidence is limited.

**Objectives:**

This study examined cross-sectional and prospective associations between total dairy, fermented dairy, and nonfermented dairy intake with *1*) the presence of elevated depressive symptoms and *2*) the risk of a future hospital discharge or outpatient diagnosis of depression.

**Methods:**

Data from 2603 Finnish men (aged 42–60 y), recruited as part of the Kuopio Ischaemic Heart Disease Risk Factor Study, were included. Multivariable logistic regression models were used to examine ORs and 95% CIs for elevated depressive symptoms (Human Population Laboratory scale ≥5 points) at baseline. Cox proportional hazards regression models were used to estimate HRs and 95% CIs between dairy categories and risk of depression diagnoses.

**Results:**

In cross-sectional analyses, fermented dairy intake in the highest (compared with lowest) tertile was associated with lower odds of having elevated depressive symptoms (adjusted OR: 0.70; 95% CI: 0.52, 0.96). Each 100-g increase in nonfermented dairy intake was associated with higher odds of having elevated depressive symptoms (adjusted OR: 1.06; 95% CI: 1.01, 1.10). During a mean follow-up time of 24 y, 113 males received a diagnosis of depression. After excluding cheese intake, higher fermented dairy intake was associated with a lower risk of depression diagnosis (adjusted HR: 0.62; 95% CI: 0.38, 1.03), which was strengthened after excluding those with elevated depressive symptoms at baseline (adjusted HR: 0.55; 95% CI: 0.31, 0.99), whereas nonfermented dairy intake in the highest tertile was associated with a 2-fold higher risk of depression (adjusted HR: 2.02; 95% CI: 1.20, 3.42).

**Conclusions:**

Fermented dairy and nonfermented dairy intake were differentially associated with depression outcomes when examined cross-sectionally and over a mean period of 24 y. These findings suggest that dairy fermentation status may influence the association between dairy intake and depression in Finnish men. The KIHD study was registered at clinicaltrials.gov as NCT03221127.

## Introduction

Depressive disorders are highly prevalent and contribute substantially to global disease burden (1). More than 260 million people are estimated to live with depression worldwide (2), which can have considerable impacts on an individual's quality of life, relationships, and functional capacity (3). Although overall diet quality has been identified as an important modifiable risk factor in both the prevention (4) and treatment of depression (5), the role of specific dietary components (i.e., dairy products) in relation to depression is less clear.

Dairy products are an important dietary source of protein, vitamins (e.g., B vitamins), micronutrients (e.g., calcium), and bioactive compounds (6). Although a large body of evidence suggests that dairy products have largely neutral or positive benefits for cardiometabolic health [e.g., cardiovascular disease (CVD) (7), type 2 diabetes (T2DM) (8)], the role of dairy intake in relation to depression is unclear (9). To date, a small number of studies have examined the association between dairy intake and depression in adults. Although a recent systematic review of observational studies found mostly no association between total dairy intake and depression, the association between intake of specific fermented and nonfermented dairy products (e.g., milk, yogurt, and cheese) and depression was inconsistent (9).

Fermented dairy products (e.g., yogurt, kefir, and certain cheeses) contain varying amounts of live microorganisms with potential probiotic effects, prebiotics, and functional metabolites (e.g., biogenics) that may confer nutritional benefits beyond that supplied by milk (10, 11). Epidemiologic studies suggest higher fermented milk intake is associated with a lower risk of conditions that share comorbidity with depression, including CVD, T2DM, and certain cancers, and improved weight maintenance and gastrointestinal health (12). Fermented dairy intake has also been shown to modify pathways central to the pathogenesis of depression, including gut microbiota composition. For example, consumption of fermented milk products has been shown to increase beneficial strains of bacteria such as *Lactobacillus* and *Bifidobacterium* (13). Furthermore, consumption of certain fermented milks has been found to have favorable effects on markers of inflammation and oxidative stress (14), and daily probiotic yogurt intake has been demonstrated to significantly reduce serum markers of inflammation (15), which is a central pathway in depression (16).

To date, a small number of studies that have explored associations between specific types of fermented (e.g., yogurt) and nonfermented (e.g., milk) dairy products in relation to depression have produced mixed findings. Although higher yogurt intake has been associated with lower odds of having depressive symptoms (17, 18), higher milk intake has been associated with a higher risk of de novo depression in postmenopausal women (19). These discrepant findings may be explained by differences in dairy fermentation status. However, no studies have collectively grouped individual dairy products by fermentation status and compared associations between total fermented dairy and total nonfermented dairy intake with depression incidence, using a physician assessment of depression (9).

Given the increased popularity of fermented foods and inclusion of dairy products within several dietary guidelines around the world (20, 21), further studies that seek to understand the role of fermented dairy intake in depression are required. Therefore, using a population with a high known level of dairy intake, this study aimed to examine associations between total dairy, fermented dairy, and nonfermented dairy intake with *1*) the presence of elevated depressive symptoms and *2*) the risk of a hospital discharge or outpatient diagnosis of depression in middle-aged and older Finnish men.

## Methods

### Study design and population

**FIGURE 1:**
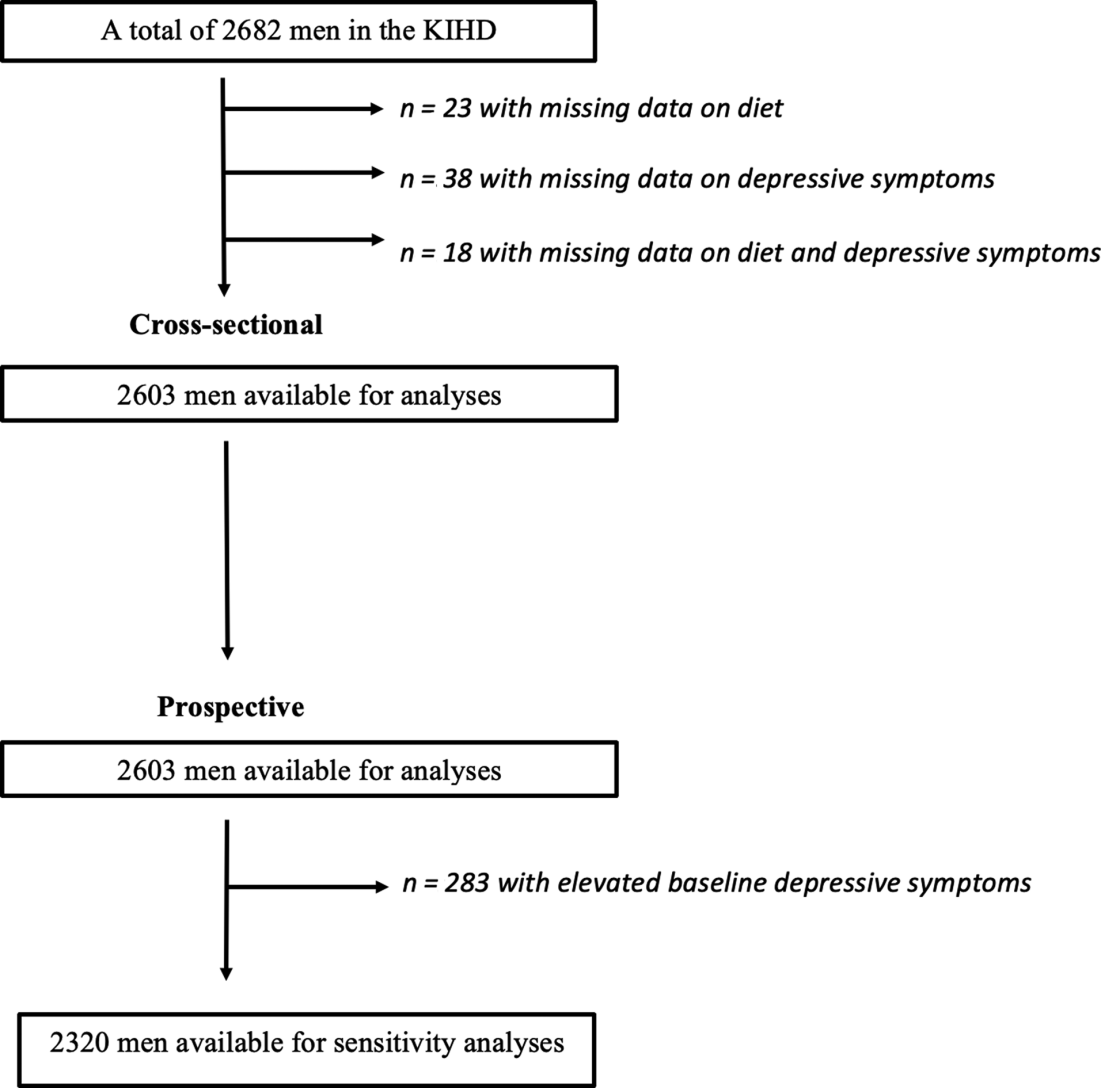
Flow diagram of the recruitment and exclusion process for the participants. KIHD, Kuopio Ischaemic Heart Disease Risk Factor Study.

The Kuopio Ischaemic Heart Disease Risk Factor Study (KIHD) is an ongoing population-based cohort study designed to investigate risk factors for CVD and other chronic diseases in middle-aged and older men from eastern Finland. Men living in the Kuopio city and neighboring rural communities were recruited in 2 cohorts (total *n* = 2682, 82.9% of those eligible). The first cohort comprised 1166 men who were 54 y old at enrollment in 1984–1986. The second independent cohort included 1516 men who were 42, 48, 54, or 60 y old at enrollment in 1986–1989 (22). Data from both cohorts were pooled for the purpose of these analyses. Data from women were not available at these time points, and therefore the present study included men only. Further details on the KIHD are described elsewhere (22). For the present study, data collected in 1984–1989 were considered baseline and used for the cross-sectional analysis. Participants with missing dietary intake data (*n* = 23) or depressive symptom data (*n* = 38) at baseline were excluded. Eighteen participants had missing data for both dietary intake and depressive symptoms, which left data from 2603 men available for analyses (see **Figure 1**). For the longitudinal analysis, all participants with available diet and depressive symptom data at baseline examinations, as well as depression diagnoses until the end of 2017, were included. The KIHD protocol was approved by the research ethics committee of the University of Kuopio and Kuopio University Hospital. Written, informed consent was obtained from all participants. Based on prior ethical approval, this present study was approved for exemption from ethical review in accordance with the National Statement on Ethical Conduct in Human Research (2007, updated 2018) Section 5.1.22 by the Deakin University Human Research Ethics Committee.

### Assessment of dairy intake (exposure)

Dietary intake was quantitatively assessed using 4-d food records at baseline, which included 3 weekdays and 1 weekend day. Participants received instructions on how to complete the food record from a nutritionist. To aid with portion-size estimates, participants were instructed to use conventional household measures and received a picture book of common foods and dishes as a reference. Upon completion, records were cross-checked by a nutritionist together with the participant to minimize reporting error. NUTRICA 2.5 (The Social Insurance Institution of Finland) was used to quantify food and nutrient composition from the food records. Food composition data in the database were primarily based on the nutritional composition of common Finnish foods. Dairy products, defined as any milk-based product, were categorized manually as either fermented dairy (e.g., sour milk, cultured buttermilk, yogurt, kefir, quark, sour cream, and fermented cheeses such as cottage, blue, edam, gouda, and Swiss) or nonfermented dairy products (e.g., milk, cream, ice cream, colostrum, and Finnish squeaky cheese, a type of baked cheese). Fermented dairy products were also analyzed separately after excluding cheese, given that cheese intake has previously been associated with adverse depression outcomes (23), and the nutrient content of cheese (e.g., fat and sodium content) and physical structure (i.e., dairy matrix) differ from that of liquid fermented dairy products (24, 25). Total dairy intake was calculated as the sum of fermented dairy and nonfermented dairy intake. Intake of total, fermented, and nonfermented dairy was first examined as continuous variables (g/d). Tertiles were then derived using data-driven cutoffs, and the median intakes for each dairy category were presented as per previous epidemiologic dietary research among large population cohorts (26). The lowest tertile was treated as the reference (tertile 1). Data were categorized into tertiles to aid with clinical interpretation of the data and enable comparability with the wider literature. For the prospective analysis, dairy categories were examined as tertiles using the a priori cutoffs established for the cross-sectional analysis.

### Assessment of depressive symptoms and depression (outcomes)

For the cross-sectional analysis, depressive symptoms were assessed using the 18-item Human Population Laboratory (HPL) depression scale (27). The HPL depression scale includes questions on mood disturbance, negative self-concept, loss of energy, problems with eating and sleeping, trouble with concentration, and psychomotor retardation or agitation (27). The scale was designed specifically for the purpose of screening general populations (27) and closely resembles well-established depression symptomology checklists such as the Centre for Epidemiological Studies Depression Scale (28). Scores were derived by assigning 1 point for each true or false answer indicative of depression. For certain items, response options of “often” or “never” were assigned 1 point where appropriate. Total possible scores ranged between 0 and 18. Baseline HPL scores were dichotomized into the absence (<5 points) or presence of elevated depressive symptoms (≥5 points), which corresponds with previous research (27, 29).

For the prospective analysis, the primary endpoint was defined as diagnosis of clinical depression by a physician. These data were obtained using computer linkage to the national hospital discharge and outpatient registers. Participants who received a hospital discharge diagnosis of a depressive disorder (from baseline until the end of 2017) or outpatient diagnosis of depression (collected from 1998 until 2017) were considered to have clinical depression. Diagnoses were made according to International Classification of Diseases (ICD) criteria and included major depression (ICD-10: F32.0–3, F33.1–3; *n* = 41); depression, an otherwise unspecified disorder (ICD-9: 2968A, ICD-10: F32.8–9, F33.9; *n* = 49); chronic depression (ICD-8: 300.41, ICD-9: 3004A, ICD-10: F34.1; *n* = 10); or adjustment disorder with depressive symptoms (ICD-9: 3090A, ICD-10: F43.2–29; *n* = 13). If the participant had multiple admissions or outpatient visits for depression during the follow-up period, the first event diagnosis was used as the endpoint.

### Assessment of covariates

Several baseline covariates were considered potential effect modifiers and selected based on their established evidence base for their association with the variables of interest (30–33). Potential confounders were selected a priori and included age; examination year; energy intake (kJ/d); fruit, berry, and vegetable intake (g/d); alcohol intake (g/wk); leisure-time physical activity (kcal/d); smoking (cigarettes packs/d multiplied by years of smoking); BMI (in kg/m^2^); marital status (married or living with a partner compared with living alone); socioeconomic status (SES); and history of CVD (yes/no) and/or T2DM (yes/no). Average alcohol intake over the past 12 mo (g/wk) was assessed with a structured quantity–frequency method using the Nordic Alcohol Consumption Inventory for drinking behavior (34). Average fruit, berry, and vegetable intakes were calculated from the baseline 4-d food diaries. Body weight and height measurements and BMI calculations were performed by the study nurse. Detailed methods for the determination of leisure-time physical activity, history of CVD and T2DM, marital status, smoking habits, and SES at baseline in this cohort have been described elsewhere (35, 36).

### Statistical analyses

The analysis plan for this study was preregistered on the Open Science Framework platform (https://osf.io/9bqwm). We first performed an exploratory cross-sectional analysis of baseline measures to examine associations between total dairy intake and subcategories of dairy intake (fermented dairy and nonfermented dairy intake) with the presence of elevated depressive symptoms. Using a priori cutoffs established from the cross-sectional analysis, we then performed a prospective confirmatory analysis of data to examine these associations in a longitudinal setting.

Differences in baseline characteristics *1*) among those in different tertiles of dairy intake (**Supplemental Table 1**) and *2*) between participants who received and did not receive a hospital discharge or outpatient diagnosis of depression during the follow-up (**Table 1**) were assessed using Mann–Whitney *U* tests or ANOVA test for continuous variables and χ^2^ tests for categorical variables. For the cross-sectional analysis, multivariable logistic regression models were used to estimate ORs and their 95% CIs for the dairy categories, adjusting for covariates. The prospective analysis was confirmatory and examined dairy tertiles using established a priori cutoffs derived from the cross-sectional analysis. Cox proportional hazards regression models were used to estimate HRs and their 95% CIs for each of the dairy categories, adjusting for covariates and baseline depressive symptom scores. The proportional hazards assumption was visually inspected by plotting the (log) cumulative hazards function. Time to first depression diagnosis (survival) curves were illustrated using Kaplan–Meier estimator of the survival function using the product limit estimator.

**TABLE 1:** Baseline characteristics of all participants and presented according to those who received or did not receive an outpatient or hospital discharge diagnosis for depression during follow-up^1^

Characteristic	All (*n* = 2603)	Diagnosis of depression (*n* = 113)	No diagnosis of depression (*n* = 2490)	*P* value
Age, y	54.3 (48.9, 54.5)	54.3 (48.5, 54.5)	54.3 (48.9, 54.5)	0.028^2^
Total dairy intake, g/d	683 (452, 927)	738 (501, 965)	682 (450, 925)	0.341^2^
Fermented dairy intake, g/d	103 (24, 282)	91 (19, 225)	106 (24, 284)	0.115^2^
Nonfermented dairy intake, g/d	473 (264, 727)	558 (371, 788)	467 (263, 724)	0.016^2^
Energy intake, kJ/d	9997 (8414, 11,650)	10,480 (8746, 12,010)	9970 (8407, 11,640)	0.102^2^
Fruit, berry, and vegetable intake, g/d	227 (138, 335)	206 (132, 303)	228 (139, 338)	0.136^2^
Alcohol, g/wk	31 (6, 91)	44 (6, 120)	31 (6, 90)	0.285^2^
Married or living with partner	2261 (87)	100 (80)	2161 (87)	0.661^3^
Cigarettes, packs/d × years of smoking	0 (0, 9)	0 (0, 10)	0 (0, 8)	0.701^2^
Leisure-time physical activity, kcal/d	85 (30, 190)	81 (36, 171)	85 (30, 191)	0.796^2^
BMI, kg/m^2^	27 (25, 29)	27 (25, 29)	27 (25, 29)	0.813^2^
Socioeconomic status, points	10 (6, 13)	10 (6, 13)	10 (6, 13)	0.531^2^
HPL depression scores at baseline	1 (0, 3)	2 (1, 4)	1 (0, 3)	<0.001^2^
History of cardiovascular disease	980 (38)	41 (36)	939 (38)	0.759^3^
History of mental illness	143 (6)	19 (17)	124 (5)	<0.001^3^

1 Values are presented as median (IQR) or *n* (%) and rounded to nearest whole number. HPL, Human Population Laboratory scale.

2 Calculated using Mann–Whitney *U* test.

3 Calculated using χ^2^ test.

Covariates were added to the models in stages, to understand the effects of covariate adjustment on model outputs. The impact of the covariates on estimated associations between the exposures of interest and outcomes was used as a basis for inclusion of the covariates in the models, not a statistical significance measure. Missing values (<2.4%) in covariates were replaced with the cohort mean. Any covariate significant at the α = 0.1 level was introduced into the model along with the dairy measurement. We performed a backward stepwise variable selection method and removed 1 covariate at a time until all covariates that remained in the model were significant at the α = 0.05 level. The potential of the covariate to be associated with both the exposure and outcome was also considered in their inclusion in the model. Model 1 included age, examination year, and energy intake. Model 2 adjusted for model 1 and alcohol intake, SES, and history of CVD. Model 3 (prospective analysis only) included model 2 and baseline HPL depression scores. Further adjustments for other covariates (e.g., BMI; fruit, berry, and vegetable intake; leisure-time physical activity; smoking; marital status; and history of T2DM) did not substantially change the estimates and therefore were not included in the final models. A model that included all covariates was also presented as a basis to compare the estimated associations from models 1–3. To minimize the risk of reverse causality, we also repeated the prospective analysis after excluding those with elevated depressive symptoms at baseline (sensitivity analysis). All statistical tests were 2-tailed and a *P* value of <0.05 was considered statistically significant. Exact *P* values and 95% CIs were reported to aid with interpretation. Statistical analyses were performed using SPSS statistical package (IBM SPSS Statistics version 27; SPSS, Inc.).

## Results

### Descriptive characteristics

In total, 2603 men were included. At baseline, elevated depressive symptoms were present in 10.9% of participants (*n* = 283). The median age of participants was 54.3 y (IQR: 48.9, 54.5), and 5.5% had a history of mental illness. The median (IQR) daily intakes of total dairy, fermented dairy, and nonfermented dairy products were 683 g/d (452, 927), 103 g/d (24, 282) and 473 g/d (264, 727), respectively. Milk contributed to 95% of nonfermented dairy intake and sour milk, which is commonly consumed as a drink during meals, accounted for 99% of fermented dairy intake.

During a mean ± SD follow-up time of 24 ± 9 y, 113 men (4.3%) were diagnosed with depression. Of these diagnoses, 62% of cases were diagnosed during a hospital admission and 38% diagnosed as outpatients. Descriptive characteristics of participants according to those with and without a depression diagnosis are presented in Table 1. Participants who received a future diagnosis of depression were younger in age, consumed more nonfermented dairy products, had higher baseline HPL depression scores, and were more likely to have a history of mental illness at baseline compared with those without a depression diagnosis at follow-up. Descriptive statistics stratified by tertiles of total dairy, fermented dairy, and nonfermented dairy intake are presented in Supplemental Table 1.

### Associations between total dairy, fermented dairy, and nonfermented dairy intake and the presence of elevated depressive symptoms

No association was observed between total dairy intake, when examined as a continuous variable, and the presence of elevated depressive symptoms at baseline (see **Supplemental Table 2**). Whereas each 100-g increase in fermented dairy intake was associated with 11% lower odds of having elevated depressive symptoms (model 2; OR: 0.89; 95% CI: 0.83, 0.96), and estimates remained significant after excluding cheese intake (model 2; OR: 0.90; 95% CI: 0.84, 0.96). In contrast, each 100-g increase in nonfermented dairy intake was associated with 6% higher odds of having elevated depressive symptoms (OR: 1.06; 95% CI: 1.01, 1.10).

When intakes of total dairy, fermented dairy, and nonfermented dairy were examined as tertiles, a similar pattern was observed. No significant association was observed between baseline total dairy intake and the presence of elevated depressive symptoms (see **Table 2**). In contrast, those in the highest (compared with lowest) tertile of fermented dairy intake had a 30% lower odds of having elevated depressive symptoms (model 2; OR: 0.70; 95% CI: 0.52, 0.96). After excluding cheese intake, this association remained relatively unchanged (model 2; OR: 0.71; 95% CI: 0.52, 0.97). In contrast, those with nonfermented dairy intake in the highest (compared with lowest) intake tertile had a 42% higher odds of having elevated depressive symptoms (model 1; OR: 1.42; 95% CI: 1.01, 1.98), but this association was attenuated after adjustment for further covariates (model 2; OR: 1.32; 95% CI: 0.93, 1.86).

**TABLE 2:** Unadjusted and multivariable logistic regression models for the cross-sectional association between total dairy, fermented dairy, and nonfermented dairy intake and the presence of elevated depressive symptoms among Finnish men

	Intake tertile			
	1	2	3	
Characteristic	Reference	OR (95% CI)	OR (95% CI)	*P*-trend
Total dairy intake				
Median (IQR) intake, g/d	346 (232, 452)	683 (611, 754)	1040 (926, 1215)	
Events/participants, *n*	98/867	92/868	93/868	
Unadjusted	1	0.93 (0.69, 1.26)	0.94 (0.70, 1.27)	0.70
Model 1^1^	1	0.93 (0.69, 1.27)	0.99 (0.70, 1.40)	0.94
Model 2^2^	1	0.86 (0.63, 1.18)	0.89 (0.62, 1.27)	0.50
Fermented dairy intake				
Median (IQR) intake, g/d	10 (0, 24)	105 (70, 154)	378 (281, 523)	
Events/participants, *n*	115/869	84/867	84/867	
Unadjusted	1	0.70 (0.52, 0.95)	0.70 (0.52, 0.95)	0.06
Model 1^1^	1	0.71 (0.53, 0.95)	0.70 (0.52, 0.95)	0.06
Model 2^2^	1	0.75 (0.55, 1.02)	0.70 (0.52, 0.96)	0.048
Fermented dairy intake (excluding cheese)				
Median (IQR) intake, g/d	0 (0, 0)	93 (46, 139)	367 (265, 509)	
Events/participants, *n*	109/871	93/876	81/856	
Unadjusted	1	0.83 (0.62, 1.11)	0.73 (0.54, 0.99)	0.06
Model 1^1^	1	0.83 (0.62, 1.11)	0.73 (0.53, 0.98)	0.06
Model 2^2^	1	0.88 (0.65, 1.19)	0.71 (0.52, 0.97)	0.03
Nonfermented dairy intake				
Median (IQR) intake, g/d	192 (118, 264)	472 (403, 549)	836 (727, 1014)	
Events/participants, *n*	83/867	97/868	103/868	
Unadjusted	1	1.19 (0.87, 1.62)	1.27 (0.94, 1.73)	0.13
Model 1^1^	1	1.24 (0.91, 1.70)	1.42 (1.01, 1.98)	0.046
Model 2^2^	1	1.21 (0.88, 1.66)	1.32 (0.93, 1.86)	0.13

1 Model 1 adjusted for age, examination year, and energy intake.

2 Model 2 adjusted for model 1 and alcohol intake, socioeconomic status, and history of cardiovascular disease.

### Associations between total dairy, fermented dairy, and nonfermented dairy intake and the risk of future depression diagnosis

**FIGURE 2:**
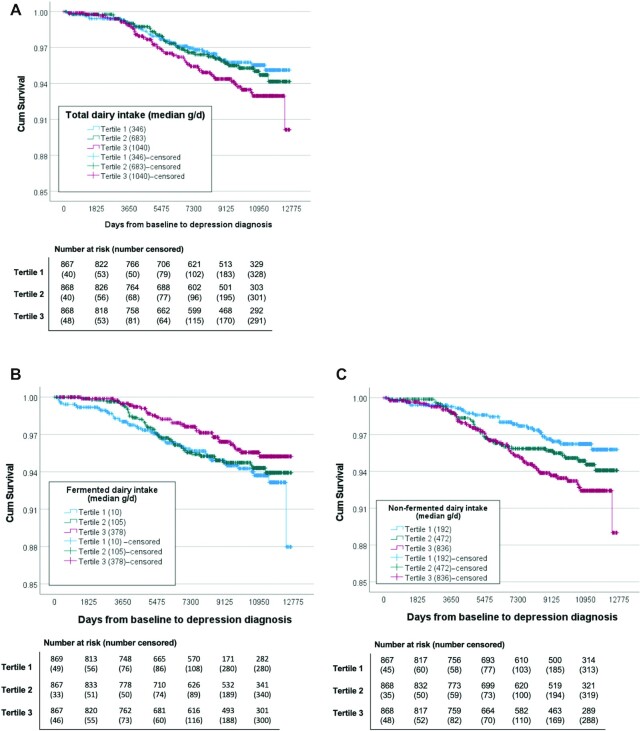
Kaplan–Meier survival curves for depression diagnosis among middle-aged and older Finnish men presented across tertiles of total dairy (A), fermented dairy (B), and nonfermented dairy (C) intake.

**Table 3** illustrates the HRs for dairy product intake using the lowest tertile as the reference. Although there was a trend for total dairy intake to be associated with a higher risk of depression diagnoses, this association was not significant in unadjusted or adjusted models. Those in the highest (compared with lowest) tertile of fermented dairy intake had a lower risk of depression diagnosis, although this association was not statistically significant (model 3; HR: 0.71; 95% CI: 0.44, 1.14). However, after excluding cheese intake, this association was slightly strengthened (model 3; HR: 0.62; 95% CI: 0.38, 1.03; *P*-trend = 0.04). In contrast, those in the highest (compared with lowest) tertile of nonfermented dairy intake had a 2-fold higher risk of depression diagnosis (model 3; HR: 2.02; 95% CI: 1.20, 3.42). In the model adjusted for all covariates, results did not substantially differ from that of the main analyses (see **Supplemental Table 3**). Kaplan–Meier survival plots for depression diagnosis among those in different tertiles of total dairy, fermented dairy, and nonfermented dairy intake can be found in **Figure 2**.

**TABLE 3:** Cox proportional hazards regression models for the association between total dairy, fermented dairy, and nonfermented dairy intake and the risk of depression diagnoses among Finnish men over a mean period of 24 y

	Intake tertile			
	1	2	3	
Characteristic	Reference	HR (95% CI)	HR (95% CI)	*P*-trend
Total dairy intake				
Median (IQR) intake, g/d	346 (232, 452)	683 (611, 754)	1040 (926, 1215)	
Events/participants, *n*	32/867	35/868	46/868	
Unadjusted	1	1.11 (0.69, 1.80)	1.51 (0.96, 2.37)	0.07
Model 1^1^	1	1.16 (0.71, 1.89)	1.61 (0.96, 2.68)	0.07
Model 2^2^	1	1.14 (0.70, 1.87)	1.63 (0.97, 2.77)	0.06
Model 3^3^	1	1.18 (0.72, 1.93)	1.67 (0.98, 2.82)	0.06
Fermented dairy intake				
Median (IQR) intake, g/d	10 (0, 24)	105 (70, 154)	378 (281, 523)	
Events/participants, *n*	43/869	41/867	29/867	
Unadjusted	1	0.87 (0.57, 1.34)	0.64 (0.40, 1.03)	0.06
Model 1^1^	1	0.87 (0.56, 1.33)	0.64 (0.40, 1.02)	0.06
Model 2^2^	1	0.92 (0.60, 1.42)	0.65 (0.41, 1.05)	0.07
Model 3^3^	1	0.96 (0.63, 1.49)	0.71 (0.44, 1.14)	0.14
Fermented dairy intake (excluding cheese)				
Median (IQR) intake, g/d	0 (0, 0)	93 (46, 139)	367 (265, 509)	
Events/participants, *n*	42/871	46/876	25/856	
Unadjusted	1	1.01 (0.66, 1.53)	0.58 (0.35, 0.95)	0.02
Model 1^1^	1	1.02 (0.67, 1.55)	0.59 (0.36, 0.96)	0.02
Model 2^2^	1	1.08 (0.71, 1.64)	0.59 (0.36, 0.97)	0.02
Model 3^3^	1	1.05 (0.69, 1.60)	0.62 (0.38, 1.03)	0.04
Nonfermented dairy intake				
Median (IQR) intake, g/d	192 (118, 264)	472 (403, 549)	836 (727, 1014)	
Events/participants, *n*	26/867	38/868	49/868	
Unadjusted	1	1.43 (0.87, 2.35)	1.95 (1.21, 3.14)	0.005
Model 1^1^	1	1.49 (0.90, 2.48)	2.11 (1.26, 3.54)	0.004
Model 2^2^	1	1.50 (0.90, 2.50)	2.11 (1.25, 3.56)	0.005
Model 3^3^	1	1.50 (0.90, 2.50)	2.02 (1.20, 3.42)	0.008

1 Model 1 adjusted for age, examination year, and energy intake.

2 Model 2 adjusted for model 1, alcohol intake, socioeconomic status, and history of cardiovascular disease.

3 Model 3 adjusted for model 2 and Human Population Laboratory scale depressive scores.

### Sensitivity analyses

As participants with depression had higher HPL depressive scores at baseline, we also performed a sensitivity analysis (see Table 4) excluding those who had initially elevated depressive symptoms at baseline (*n* = 283). In this sensitivity analysis (*n* = 2320 total, *n* = 87 diagnosed depression), the HRs for depression risk among those in the highest tertile of both total dairy intake (model 2; HR: 1.62; 95% CI: 0.90, 2.94) and nonfermented dairy intake (model 2; HR: 2.19; 95% CI: 1.20, 4.01) did not change substantially from those presented in the main analysis. The association between total fermented dairy intake and depression was slightly strengthened, however this association remained not statistically significant (model 2, HR: 0.60; 95% CI: 0.35, 1.05). Whereas the HRs for depression risk among those in the highest tertile (compared with lowest tertile) of fermented dairy intake (excluding cheese intake) reached statistical significance (model 2; HR: 0.55; 95% CI: 0.31, 0.99).

**TABLE 4:** Cox proportional hazards regression models for the associations between total dairy, fermented dairy, and nonfermented dairy intake and the risk of depression diagnoses among Finnish men over a mean period of 24 y, after excluding those with elevated Human Population Laboratory scale depressive symptom scores at baseline (sensitivity analysis)

	Intake tertile			
	1	2	3	
Characteristic	Reference	HR (95% CI)	HR (95% CI)	*P*-trend
Total dairy intake				
Median (IQR) intake, g/d	346 (232, 452)	683 (611, 754)	1040 (926, 1215)	
Events/participants, *n*	25/769	24/776	38/775	
Unadjusted	1	0.97 (0.56, 1.71)	1.59 (0.96, 2.63)	0.06
Model 1^1^	1	0.99 (0.56, 1.76)	1.59 (0.90, 2.83)	0.10
Model 2^2^	1	0.98 (0.55, 1.75)	1.62 (0.90, 2.94)	0.10
Fermented dairy intake				
Median (IQR) intake, g/d	10 (0, 24)	105 (70, 154)	378 (281, 523)	
Events/participants, *n*	32/754	34/783	21/783	
Unadjusted	1	0.94 (0.58, 1.53)	0.61 (0.35, 1.06)	0.06
Model 1^1^	1	0.93 (0.57, 1.51)	0.59 (0.34, 1.03)	0.05
Model 2^2^	1	0.98 (0.60, 1.58)	0.60 (0.35, 1.05)	0.05
Fermented dairy intake (excluding cheese)				
Median (IQR) intake, g/d	0 (0, 0)	93 (46, 139)	367 (265, 509)	
Events/participants, *n*	31/762	38/783	18/775	
Unadjusted	1	1.11 (0.69, 1.78)	0.55 (0.31, 0.99)	0.02
Model 1^1^	1	1.12 (0.70, 1.81)	0.55 (0.31, 0.99)	0.02
Model 2^2^	1	1.17 (0.73, 1.90)	0.55 (0.31, 0.99)	0.02
Nonfermented dairy intake				
Median (IQR) intake, g/d	192 (118, 264)	472 (403, 549)	836 (727, 1014)	
Events/participants, *n*	19/784	30/771	38/765	
Unadjusted	1	1.57 (0.88, 2.78)	2.13 (1.23, 3.69)	0.007
Model 1^1^	1	1.58 (0.88, 2.84)	2.16 (1.19, 3.93)	0.01
Model 2^2^	1	1.61 (0.89, 2.89)	2.19 (1.20, 4.01)	0.01

1 Model 1 adjusted for age, examination year, and energy intake.

2 Model 2 adjusted for model 1, alcohol intake, socioeconomic status, and history of cardiovascular disease.

## Discussion

In this population-based cross-sectional and prospective study, we examined whether total dairy, fermented dairy, and nonfermented dairy intakes were associated with the presence of elevated depressive symptoms or risk of depression diagnosis in Finnish men. Although no significant association was observed for total dairy intake, the direction of association differed based on dairy fermentation status. Higher fermented dairy intake at middle age was associated with lower odds of having elevated depressive symptoms and a lower risk of depression diagnosis after excluding cheese intake. In contrast, higher nonfermented dairy intake at middle age was associated with higher odds of having elevated depressive symptoms and a 2-fold higher risk of depression diagnosis over a mean period of 24 y. This study adds to limited evidence on the association between fermented and nonfermented dairy intake in relation to depression incidence.

Our findings suggest that fermented dairy and nonfermented dairy intake may be differentially associated with depression. This finding is in line with prior research which observed a protective association between fermented dairy intake, but not nonfermented dairy intake, and health outcomes (e.g., CVD) (26). Importantly, cross-sectional findings revealed that higher fermented dairy intake was inversely associated with having elevated depressive symptoms. Concordant with our findings, 3 prior studies have found that higher yogurt intake was associated with lower odds of having depressive symptoms or a lower risk of depression (17, 18, 37), whereas a cross-sectional study in Chinese adults found that high frequency of yogurt intake (≥2 times/d), reported by a small subset of participants, was associated with increased depressive symptoms (38). This discrepant finding may be due to differences in overall dairy intake between study populations, particularly given total dairy intake in our population was high relative to other studies. Furthermore, these contrasting results could be explained by other components within fermented dairy products. For example, unlike sour milk, which is likened to buttermilk and accounted for the greatest proportion of fermented dairy intake in our study, yogurt is commonly sweetened with added sugars, which have been linked to increased depressive symptoms (39, 40).

Interestingly, in the present study, higher fermented dairy intake was significantly associated with a lower risk of depression diagnosis only after excluding cheese intake. Higher cheese intake has previously been associated with adverse depression outcomes, including a higher risk of severely depressed mood in men (23). The nutritional composition of cheese differs from that of liquid fermented dairy products, such as sour milk, kefir, and buttermilk (25). For example, cheese has a higher sodium content, which has been linked to a higher risk of CVD, which may thereby increase predisposition to developing depression (41). Furthermore, cheese is more commonly consumed as part of nutrient-poor, energy-dense foods (e.g., pizzas, burgers), which, as part of a broader unhealthy dietary pattern, may increase depression risk (4). Consequently, including cheese alongside liquid fermented dairy products, may have attenuated associations.

A surprising finding was that nonfermented dairy intake was associated with a more than 2-fold higher risk of depression diagnoses. This association remained significant after adjustment for various lifestyle and dietary factors. Consumption of milk accounted for the greatest proportion of nonfermented dairy intake in the present study. In line with our findings, prior research has observed a higher risk of de novo clinical depression among postmenopausal women with high milk intake (≥250 mL/d) (42), whereas other studies have reported no or inverse associations between milk intake and depression outcomes (18, 43, 44). These discrepant findings may be explained by differences in study populations, levels of milk intake, or other components within nonfermented dairy products, such as fat content. Although not all studies have examined differences between dairy products that differed in fat content, a recent cross-sectional study in US adults found that intake of whole fat milk, but not low-fat or skim milk, was inversely associated with depressive symptoms (17). As such, it is possible that fermentation status alone may not fully account for the observed findings between nonfermented dairy intake and depression risk.

Several plausible mechanisms may explain our observed findings. First, fermented dairy intake may lower depression risk by modifying pathways associated with depression, including oxidative stress and inflammation (16, 45). For example, fermented dairy intake has been inversely associated with urine markers of oxidative stress (41), and intake of certain probiotic fermented milks has been shown to have beneficial effects on markers of inflammation and oxidative stress (14). The gut microbiome has also been increasingly implicated in the pathogenesis of mental disorders, including depression (46). Diet is now recognized as a key component in shaping overall composition of the gut microbiota (47), and fermented dairy products, such as yogurt and kefir, have been reported to increase the abundance of beneficial bacteria (*Lactobacillus* and *Bifidobacterium*) (13). Second, given higher fermented dairy intake has been associated with a lower risk of CVD (48), fermented dairy products may also have indirect benefits for mental health, through improved physical health outcomes. Third, given the bidirectional relation that exists between diet and depression, it is also plausible that individuals with depression may have changed their dietary habits regarding dairy intake. For example, symptoms of depression have been associated with higher intakes of saturated fat and sugar, which are found in higher quantities in certain dairy foods (e.g., nonfermented products such as ice cream) (49, 50). Fourth, beneficial components within the fermented food matrix may protect against potentially deleterious compounds that naturally occur in milk products, such as dietary galactose (D-galactose). D-galactose is produced upon enzymatic digestion of lactose within milk products and is present in lower quantities in fermented dairy products due to the action of specific lactic acid bacteria (51). D-galactose has been used in experimental models to accelerate aging (52) and has been shown to increase oxidative stress in the brain (53), which may negatively affect depression risk. Last, our finding of the higher risk for depression among those who consumed more nonfermented dairy products could be explained by higher A1 β-casein intake, a protein found within conventional dairy products. Finland has a relative high milk intake compared with other countries (54) and is also among countries with the highest A1 β-casein intake per capita (55). Intake of conventional milk containing A1 β-casein has been shown to increase inflammation and oxidative stress in small clinical trials (56, 57). These observations are largely thought to be due to the action of β-casomorphin-7 (BCM-7), a bioactive peptide with opioid-like effects released upon gastrointestinal digestion and processing of milk products (58, 59). However, BCM-7 is degraded during the fermentation process and is found in lower amounts in yogurt and cheese when compared with milk, which may in part explain the observed findings (60, 61).

Strengths of this study include the use of detailed dietary assessment methods obtained via 4-d food records. This method is less prone to recall bias and captures in-depth dietary intake data with respect to the types of dairy products consumed compared with other dietary assessment methods (e.g., FFQs). This study also considered a broader range of fermented dairy products (e.g., sour milk, cultured buttermilk, yogurt, kefir, quark, sour cream, and fermented cheeses such as cottage, blue, edam, gouda, and Swiss) than previously considered by other studies (e.g., yogurt and cheese) (9).

While prior research has predominantly assessed depressive symptoms using self-report measures (9), the present study assessed depression diagnosis by linkage to both national hospital and outpatient registries, a method that has been previously substantiated for obtaining valid diagnostic data (62). Furthermore, numerous sociodemographic and lifestyle factors that are known to be associated with both diet and depression were measured and adjusted for in analyses, including energy intake. For robustness, we also conducted a sensitivity analysis to address residual confounding by the presence of elevated depressive symptoms at baseline, which reduces the likelihood of results being due to reverse causality.

Limitations of this study include the measurement of dietary data at baseline examinations only. Although fermented dairy foods have long been consumed as part of the traditional Finnish diet, intakes of fermented dairy products such as sour milk have decreased, whereas intakes of cheese and yogurt have increased during the past decades (63). However, although dairy intake at baseline may not accurately reflect intake over the study entirety, other population-based studies in men found overall diet quality to be highly consistent at baseline and 15-y follow-up assessments (64). While the use of 4-d food records may not reflect usual long-term dietary intake, seasonal variations in dairy intake is not likely very large, especially when compared with other food groups (e.g., fruit, berries, and vegetables). Further, given dairy products are typically consumed daily or almost daily, 4 d may be long enough to capture typical dairy intakes. Depressive symptoms at baseline were also assessed using the self-report HPL depression scale. Underreporting of symptoms by individuals has been observed using self-report depression assessment scales; therefore, measures of depressive symptoms at baseline may be conservative (65). Furthermore, observation from only 1 time point does not provide information of those who may have been depressed before or will be later, given depression is usually a recurrent illness. We also did not have data on the possible treatment or length of the current depressive episode or treatments, such as antidepressant data. As for the prospective analyses, although the prevalence of depression among men (4.8%) is similar to that of other population-based Finnish studies (6.8%) (66), the assessment of depression using physician diagnosis may mean that only moderate to severe cases of depression were captured and therefore our overall number of cases was relatively low. Whilst we adjusted for numerous potential confounders, it cannot be ignored that residual confounding due to unmeasured or poorly measured confounders may be present. Lastly, caution should be used when generalizing findings to women and other populations in which dairy intake patterns may differ.

In this population-based study among Finnish men, we found that fermented dairy and nonfermented dairy intakes were differentially associated with depression outcomes. These findings suggest that dairy fermentation status may be an important factor in the association between dairy intake and depression. Given the widespread intake of dairy products and recommendations to consume dairy products by numerous dietary guidelines, such findings hold significance for public health (20, 21). If corroborated by future research, including moderate amounts of fermented dairy products (e.g., sour milk, kefir, yogurt) while limiting intake of nonfermented dairy products (e.g., milk) could form part of dietary recommendations for the prevention of depression. To confirm findings observed in this study, prospective studies that include repeated measures of diet and are conducted in different populations and sexes are required. Other components in fermented and nonfermented dairy products that may drive these observed associations should also be explored.

## Supplementary Material

nxac128_Supplemental_File

## Data Availability

Data described in this manuscript will not be made available because it contains sensitive personal data of the participants, which cannot be completely anonymized.

## Notes

The authors reported no funding received for this study.

Author disclosures: MH is supported by an Australian Rotary Health PhD Scholarship. EH is funded by the Australian National Health and Medical Research Council (No. 1156909). TR has received grants, fellowships, and research support from University of the Sunshine Coast, Australian Postgraduate Awards, Fernwood Foundation, and Be Fit Food. TR received consultancy, honoraria, and travel funds from Oxford University Press, the University of Melbourne, the University of Sydney, Bond University, University of Southern Queensland, Dietitians Association of Australia, Nutrition Society of Australia, The Royal Australian and New Zealand College of Psychiatrists, Academy of Nutrition and Dietetics, Black Dog Institute, Australian Rotary Health, Australian Disease Management Association, Department of Health and Human Services, Primary Health Networks, Positive Schools, Barwon Health, West Gippsland Healthcare Group, Central West Gippsland Primary Care Partnership, Parkdale College, City of Greater Geelong, and Global Age. FNJ has received competitive grant/research support from the Brain and Behaviour Research Institute, the National Health and Medical Research Council (NHMRC), Australian Rotary Health, the Geelong Medical Research Foundation, the Ian Potter Foundation, and University of Melbourne; industry support for research from Meat and Livestock Australia, Woolworths Limited, the A2 Milk Company, and Be Fit Foods; philanthropic support from the Fernwood Foundation, Wilson Foundation, the JTM Foundation, the Serp Hills Foundation, the Roberts Family Foundation, and the Waterloo Foundation; and travel support and speakers honoraria from Sanofi-Synthelabo, Janssen Cilag, Servier, Pfizer, Network Nutrition, Angelini Farmaceutica, Eli Lilly, and Metagenics. FNJ has written 2 books for commercial publication. HM is the recipient of a NHMRC-ARC Dementia Training Fellowship. AR is a recipient of Postdoctoral Research Fellowship from Faculty of Health, Deakin University, Australia, and has received VTR research funding from Kuopio University Hospital, Finland. She holds a university lecturer's position at the University of Eastern Finland. AR has received travel or speakers honoraria funds from Nutrition Society of Australia, Eastern Finland Medicine Association, University of Turku, and the Association of Clinical and Public Health Nutritionists in Finland. The Food and Mood Centre has received research support from the A2 Milk Company, outside the submitted work. All other authors report no conflicts of interest.

Supplemental Tables 1–3 are available from the “Supplementary data” link in the online posting of the article and from the same link in the online table of contents at https://academic.oup.com/jn/.

Abbreviations used: BCM-7, β-casomorphin-7; CVD, cardiovascular disease; HPL, Human Population Laboratory scale; ICD, International Classification of Diseases; KIHD, Kuopio Ischaemic Heart Disease Risk Factor Study; SES, socioeconomic status; T2DM, type 2 diabetes mellitus.
