# Supplementary material for: Nonfermented Dairy Intake, but Not Fermented Dairy Intake, Associated with a Higher Risk of Depression in Middle-Age and Older Finnish Men
Source: J Nutr. 2022 Jun 2;152(8):1916–26. doi: 10.1093/jn/nxac128 (PMC9361734; doi:10.1093/jn/nxac128)
Supplement: nxac128_Supplemental_File [file nxac128_supplemental_file.docx]

Non-Fermented Dairy Intake, But Not Fermented Dairy Intake, Associated with a Higher Risk of Depression in Middle-Age and Older Finnish Men. Hockey et al. “Online Supplementary Material”

**Supplementary Table 1*.*** *Baseline characteristics of participants stratified by tertiles of total dairy, fermented dairy, and non-fermented dairy intake^1^*

|  | Total dairy intake | | | Fermented dairy intake | | | Non-fermented dairy intake | | |
| --- | --- | --- | --- | --- | --- | --- | --- | --- | --- |
|  | **Tertile 1 (346g/d^2^)** | **Tertile 2 (683g/d^2^)** | **Tertile 3 (1040g/d^2^)** | **Tertile 1 (10g/d^2^)** | **Tertile 2 (105g/d^2^)** | **Tertile 3 (378g/d^2^)** | **Tertile 1 (192g/d^2^)** | **Tertile 2 (472g/d^2^)** | **Tertile 3 (836g/d^2^)** |
| Age, years | 54.3 (48.5, 54.5) | 54.3 (54.2, 54.7) | 54.3 (54.2, 54.5) * | 54.3 (48.8, 54.6) | 54.3 (48.8, 54.5) | 54.3 (54.2, 54.6) * | 54.3 (48.8, 54.6) | 54.3 (54.1, 54.5) | 54.3 (54.2, 54.5) |
| Total energy, kJ/d | 8739 (7412, 10230) | 9646 (8524,  11080) | 11520 (10180, 13300) * | 9746 (8181, 11500) | 9886 (8358, 11500) | 10270 (8729,  11850) * | 8910 (7585, 10350) | 9807 (8476,  11270) | 11310 (9801, 13030) * |
| Fruit, berry, and vegetable intake (g/d) | 235 (147, 344) | 223 (131, 339) | 224 (136, 325) | 204 (121, 308) | 232 (147, 340) | 250 (153, 355) * | 240 (146, 358) | 235 (143, 335) | 207 (126, 309) * |
| Alcohol, g/week | 44 (9, 112) | 29 (6, 86) | 22 (3, 80) * | 30 (6, 96) | 33 (7, 91) | 31 (5, 89) | 41 (9, 105) | 30 (6, 88) | 24 (4, 83) |
| Marital status: married or living with partner (%) | 89 | 87 | 85 * | 85 | 88 | 88 * | 90 | 86 | 84 * |
| Cigarettes, packs/d x years of smoking | 0 (0, 5) | 0 (0, 11) | 0 (0, 17) * | 0 (0, 19) | 0 (0, 8) | 0 (0, 1) * | 0 (0, 0) | 0 (0, 8) | 0 (0, 20) * |
| Leisure-time physical activity, kcal/d | 103 (39, 218) | 83 (30, 198) | 71 (22, 168) * | 74 (23, 170) | 92 (37, 190) | 87 (30, 207) | 98 (38, 212) | 90 (35, 207) | 70 (22, 160) * |
| BMI, kg/m^2^ | 26 (25, 29) | 27 (25, 29) | 27 (24, 29) | 27 (24, 29) | 26 (25, 29) | 27 (25, 29) | 26 (25, 29) | 27 (25, 29) | 27 (24, 29) |
| SES, points | 8 (4, 12) | 10 (6, 13) | 11 (8, 14) * | 10 (6, 13) | 8 (5, 12) | 10 (6, 13) * | 8 (4, 12) | 10 (5, 13) | 11 (7, 14) * |
| HPL depression scores | 1 (0, 3) | 1 (0, 3) | 1 (0, 3) | 1 (0, 3) | 1 (0, 3) | 1 (0, 3) | 1 (0, 3) | 1 (0, 3) | 2 (0, 3) * |
| History of CVD (%) | 36 | 39 | 38 | 38 | 36 | 40 | 37 | 38 | 38 |
| History of mental illness (%) | 6 | 5 | 6 | 7 | 5 | 5 * | 5 | 5 | 7 |

^1^Values presented as median (IQR) or n (%). ^2^Median intake g/d. **P-*trend across tertiles ≤ 0.05. *P-*trend was assessed with one-way ANOVA for continuous variables and χ2 test for categorical variable. Figures rounded to nearest whole number. BMI, body mass index; CVD, cardiovascular disease; HPL, Human Population Laboratory scale (baseline); SES, socioeconomic status

**Supplementary Table 2.** *Unadjusted and multivariable logistic regression models for the cross-sectional associations between total dairy, fermented dairy, and non-fermented dairy intake (as continuous variables) and the presence of elevated depressive symptoms*

|  | | OR (95% CI) | | P-value | |
| --- | --- | --- | --- | --- | --- |
| *Total dairy intake* | | | | | |
| Unadjusted | | 1.00 (0.97-1.04) | | 0.89 | |
| Model 1^1^ | | 1.01 (0.97-1.06) | | 0.51 | |
| Model 2^2^ | | 1.00 (0.96-1.04) | | 0.99 | |
| *Fermented dairy intake* | | | | | |
| Unadjusted | | 0.90 (0.84-0.96) | | 0.002 | |
| Model 1^1^ | | 0.90 (0.84-0.96) | | 0.002 | |
| Model 2^2^ | | 0.89 (0.83-0.96) | | <0.001 | |
| *Fermented dairy intake (excluding cheese)* | | | | | |
| Unadjusted | | 0.91 (0.85-0.97) | | 0.004 | |
| Model 1^1^ | | 0.91 (0.85-0.97) | | 0.004 | |
| Model 2^2^ | | 0.90 (0.84-0.96) | | 0.002 | |
| *Non-fermented dairy intake* | | | | | |
| Unadjusted | | 1.04 (1.01-1.08) | | 0.027 | |
| Model 1^1^ | | 1.07 (1.02-1.11) | | 0.003 | |
| Model 2^2^ | | 1.06 (1.01-1.10) | | 0.012 | |

^1^Model 1 adjusted for age, examination year and energy intake

^2^Model 2 adjusted for model 1 and alcohol intake, socioeconomic status, and history of cardiovascular disease

**Supplementary Table 3.** *Cox proportional hazards regression models for the association between total dairy, fermented dairy, and non-fermented dairy intake and the risk of depression diagnoses, adjusted for all covariates^1^*

|  | Intake tertile | | | | |  |
| --- | --- | --- | --- | --- | --- | --- |
|  | 1 | 2 | | 3 | |  |
|  | HR | HR (95% CI) | | HR (95% CI) | | P-trend |
|  |  |  |  |  |  |  |
| Total dairy intake | 1 | 1.16 (0.70-1.90) | | 1.61 (0.95-2.74) | | 0.07 |
| Fermented dairy intake | 1 | 0.98 (0.63-1.51) | | 0.74 (0.46-1.19) | | 0.19 |
| Fermented dairy intake (excluding cheese) | 1 | 1.06 (0.70-1.62) | | 0.64 (0.39-1.06) | | 0.05 |
| Non-fermented dairy intake | 1 | 1.49 (0.89-2.48) | | 1.94 (1.14-3.31) | | 0.02 |

^1^Model adjusted for age, examination year, energy intake, alcohol intake, fruit, berry, and vegetable intake, body mass index, socioeconomic status, smoking, marital status, HPL depressive scores, history of cardiovascular disease and diabetes.

HPL, Human population laboratory scale
